# Fatal Hemorrhagic Septicemia in Common Guitarfish (*Rhinobatos rhinobatos*) Caused by *Photobacterium damselae* Subsp. *damselae* in a Controlled Environment

**DOI:** 10.1111/jfd.70066

**Published:** 2025-10-04

**Authors:** Giorgia Bignami, Teresa Pirollo, Perla Tedesco, Monica Caffara, Maria Letizia Fioravanti, Elena Campesi, Cristiano Da Rugna, Stefano Gridelli, Francesco Quaglio, Andrea Gustinelli

**Affiliations:** ^1^ Department of Veterinary Medical Sciences University of Bologna Bologna Italy; ^2^ Acquario di Cattolica Cattolica Italy; ^3^ Department of Comparative Biomedicine and Nutrition Università di Padova Padova Italy

**Keywords:** aquarium management, common guitarfish *Rhinobathos rhinobathos*, hemorrhagic septicemia, *Photobacterium damselae* susbp. *damselae*

## Abstract

Elasmobranchs, including sharks and rays, are commonly housed in public aquariums due to their ecological significance and educational value. The common guitarfish (*Rhinobatos rhinobatos*), currently listed as ‘Critically Endangered’ by the IUCN, is particularly susceptible to population declines due to overfishing and bycatch. While generally considered robust, individuals in captivity may experience stress‐related health issues, increasing their susceptibility to infectious diseases. This study investigates a mortality event affecting three 20‐year‐old guitarfish kept in a public aquarium. The fish exhibited respiratory distress and ataxia before sudden death. Necropsy findings included external hemorrhages and severe hemorrhagic enteritis. Bacteriological analyses identified *Photobacterium damselae* subsp. *damselae* in all specimens through MALDI‐TOF and PCR sequencing, while parasitological tests and RT‐PCR for *Betanodavirus* were negative. Histopathology revealed bacterial aggregates in the gills, heart and kidney, consistent with systemic bacterial septicemia. *P. damselae* subsp. *damselae* is an opportunistic marine pathogen known to cause hemorrhagic septicemia in various fish species. This case represents the first documented occurrence of fatal *P. damselae* subsp. *damselae* septicemia in captive guitarfish. Understanding the impact of infectious diseases in confined environments is essential for improving the health management of endangered elasmobranchs in aquariums and conservation programs.

The guitarfish *Rhinobathos rhinobatos* is a cartilaginous fish of the family Rhinobatidae known for its distinctive appearance, resembling a fusion of sharks and rays. Due to intense fishing pressure, this species is severely endangered as a result of bycatch (Basusta et al. 2008) and was included in appendix II of the Convention on the Conservation of Migratory Species of Wild Animals (CMS) in 2017. Consequently, maintaining these animals in aquaria or zoological parks represents a potential strategy to preserve species that might otherwise face extinction (Taskin et al. 2022). Among the various issues that can arise in aquarium environments, bacterial diseases represent a major concern, often caused by opportunistic pathogens. In particular, several Gram‐negative bacteria, such as *Vibrio* spp. and members of the Flavobacteriaceae, which are ubiquitous in aquatic environments, can trigger severe superficial or systemic infections when predisposing conditions occur (Fioravanti and Florio 2017). Moreover, there is limited knowledge regarding the diseases affecting this species (Bağci et al. 2023).

*Photobacterium damselae* , a Gram‐negative marine pathogen, includes two different subspecies: *P. damselae* subsp. *piscicida* (formerly *Pasteurella piscicida*, agent of fish Pastuerellosis) and *P. damselae* subsp. *damselae* (formerly *Vibrio damselae*) (Catanese and Grau 2023).

The present case report represents the first description of *P. damselae* subsp. *damselae* as a cause of enteric septicaemia in captive guitarfish kept in a confined environment.

In December 2023, in an aquarium tank of 56,000 L of capacity containing various cartilaginous and bony fish, three guitarfish ( *R. rhinobatos* ) died suddenly after exhibiting nonspecific symptoms for 1 day, including respiratory distress and ataxia. No evidence of significant environmental changes within the tank was observed, with parameters such as water temperature of 24.6°C, pH of 8.4, pO_2_ of 89% and salinity of 32.4 ppt, all considered within normal ranges. The three guitarfish were over 20 years old and consisted of two females and one male, with an average weight of 21.6 kg (14.5 kg for the male, 22.5 and 28 kg for the females, respectively). These animals had been kept in the aquarium tank for more than 15 years. After death, the elasmobranchs were immediately transported in cooling conditions (4°C) to the Fish Pathology Unit of the Department of Veterinary Medical Sciences, University of Bologna for diagnostic purposes. Necropsy, as well as bacteriological, histological, virological and parasitological examinations were performed.

Bacteriological examination was performed by inoculating samples from the brain, kidney, spleen, eye, heart, stomach, intestine and gonads onto TSA (Tryptone Soy Agar) supplemented with 1.5% NaCl and TCBS (Thiosulfate‐citrate‐bile salts‐sucrose agar). All plates were incubated at 25°C ± 1°C for 48 h. For preliminary identification of the isolates, MALDI‐TOF was used, and simultaneously DNA from pure colonies was extracted by the boiling method to identify the subspecies of *P. damselae* . A multiplex‐PCR reaction was performed with primers Ure‐5′/Ure‐3′, specific for subsp. *damselae*, and the primers 76a/76b for amplification of subsp. *piscicida*, following Osorio et al. (2000). Additionally, kidney and spleen imprints were stained with Ziehl‐Neelsen. For histology, portions of gills, heart, brain, liver, spleen, kidney, stomach, intestine and gonads were preserved in 10% neutral buffered formalin. Sections of 5 μm were stained using Haematoxylin and Eosin (HE). Regarding the virological analysis, brain samples were pooled, and RT‐PCR for *Betanodavirus* was carried out according to the protocol described by Bovo et al. (2011). The parasitological examination was carried out by microscopical observation of fresh mounts from skin, gills and visceral organs.

During the post‐mortem examination, the pathological findings observed were non‐specific but overall suggestive of acute systemic septicemia. This condition was consistently observed in all three subjects and was characterised by severe petechial and ecchymoses, particularly along the ventral area, as well as significant haemorrhages in the ventral region of the body (Figure 1a) and, to a lesser extent, on the fins. One female showed an erosive lesion of the dorsal fin (Figure 1b) with the presence of bacterial aggregates referable to Flavobacteriaceae, evident under a light microscope.

**FIGURE 1:**
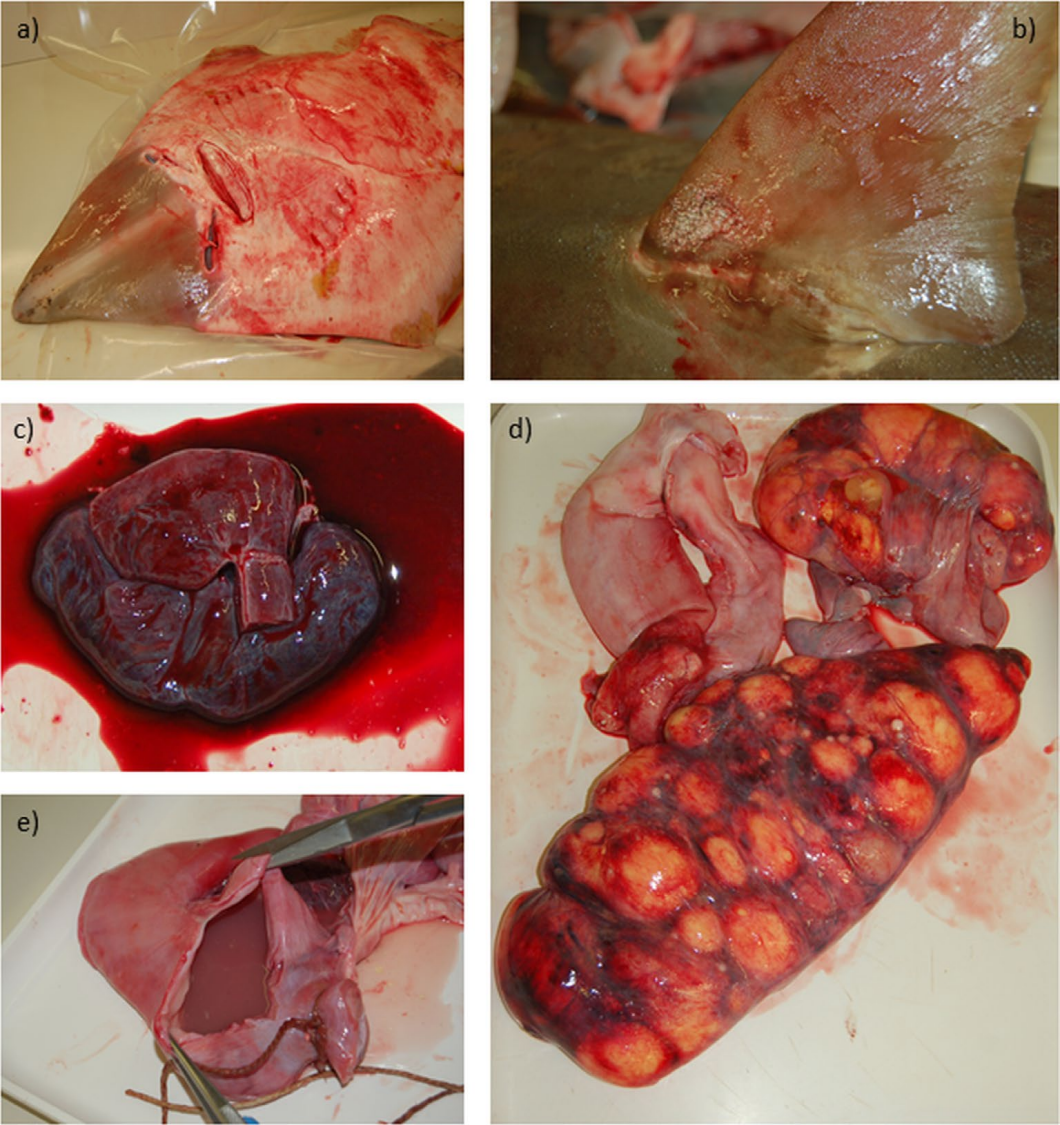
Gross lesions detected in *R. rhinobatos* . (a) Petechial lesions and ecchymoses are mainly located on the skin, particularly along the ventral area. (b) Erosive lesion at the dorsal fin level. (c) Severe congestion of the heart and (d) gonads. (e) Detail of enlarged stomach with mucous content.

In the visceral organs, severe enteritis with prominent blood clots was observed, along with marked congestion at the spiral valve, congestion and haemorrhages in the liver, heart and gonads (Figure 1c,d). The male exhibited haemorrhagic or mucous content in the stomach and intestine (Figure 1e).

The bacteriological examination on all tested media showed abundant growth of a single colony morphotype in all inoculated organs. On TCBS, green colonies were observed, which were identified as the opportunistic bacterium *P. damselae* . The MALDI‐TOF analysis assigned all the colonies as *P. damselae* subsp. *damselae*, with a score above 2.20. The identification was also confirmed by PCR. Virological and parasitological exams were negative.

Histopathological examination of the gills revealed severe lamellar congestion, oedema, necrosis with cell sloughing, and the presence of debris, along with widespread bacterial aggregates (Figure 2a,b). The stomach showed focal mucosal necrosis with erosion, inflammation with lymphocytic infiltrate in the lamina propria and submucosa (Figure 2c). Mild hepatic congestion and an increased presence of melanomacrophages (Figure 2d), together with focal splenic necrosis and bacterial aggregates within the lumen of the vessels, were detected.

**FIGURE 2:**
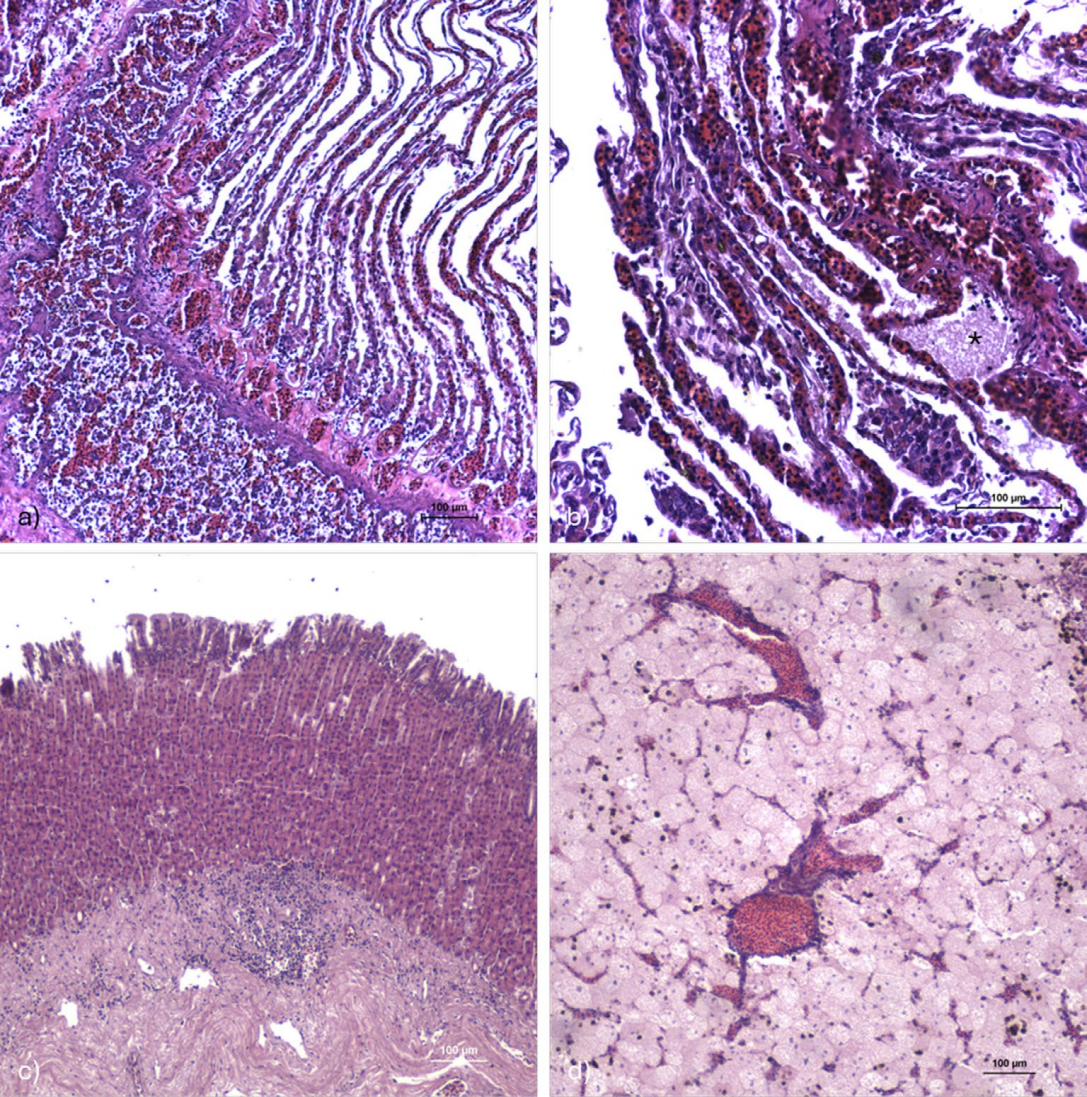
Histological section of the gills, stomach and liver of an infected guitarfish (HE): (a) Gills showing oedema, epithelial sloughing and necrosis (scale bar = 100 μm). (b) Detail of a secondary lamellae with congestion and presence of bacterial aggregate (asterisk) (scale bar = 100 μm). (c) Inflammation with lymphocytic infiltrate in the lamina propria and submucosa of the stomach (scale bar = 100 μm). (d) Detail of a hepatic congestion (scale bar = 100 μm).

The heart exhibited congestion of the coronary arteries (Figure 3a), diffuse necrosis, severe atrial myocardial haemorrhages and vascular congestion. Focal lymphocytic infiltrates with eosinophilic granular cells and giant cells were observed, as well as lipid‐like droplets and abnormal amorphous intracellular retention of lipofuscin‐like material.

**FIGURE 3:**
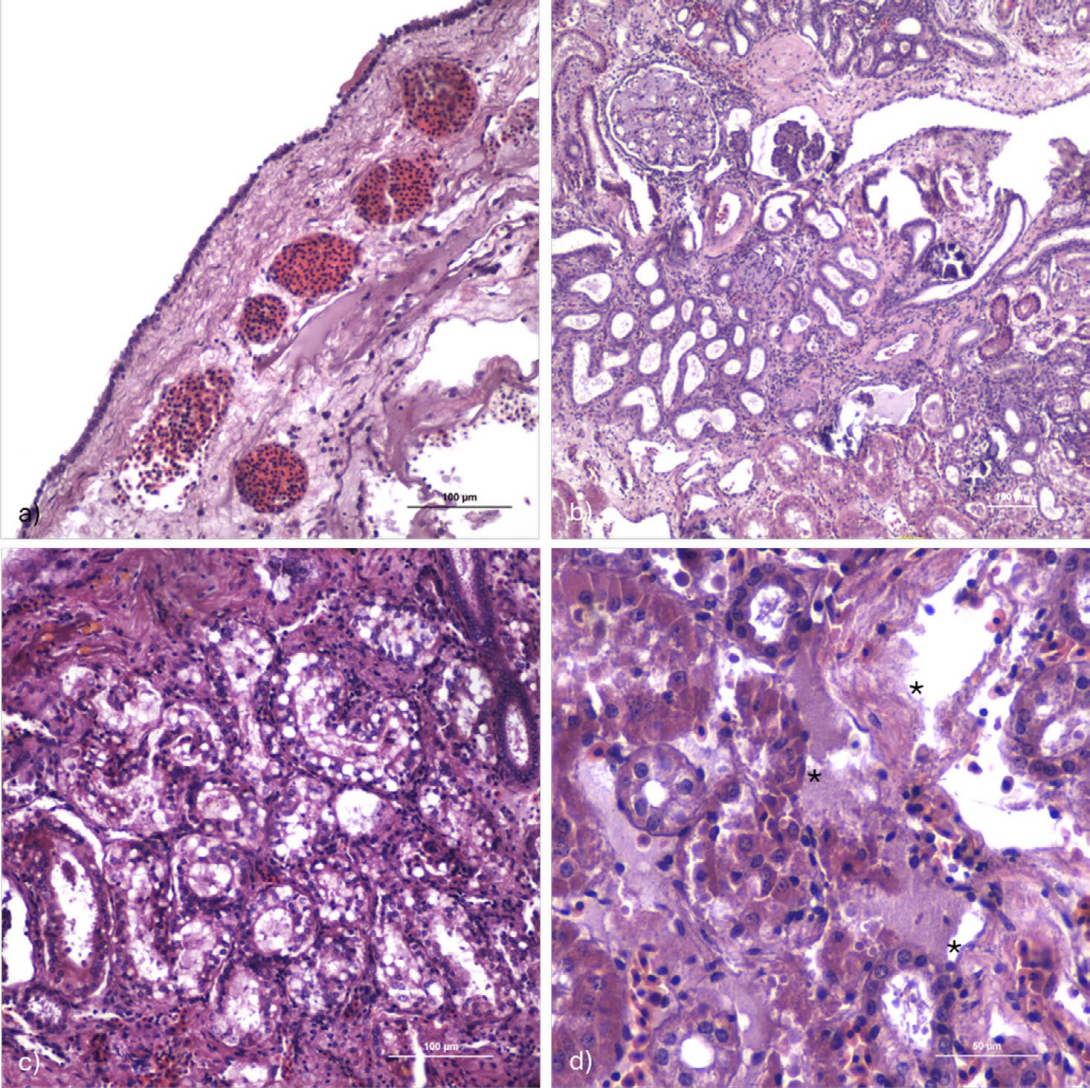
Histological section of the heart and kidney of an infected guitarfish (HE): (a) Congestion of coronaries (scale bar = 100 μm). (b) Kidney with diffuse tubular ectasia, some of which contain basophilic matter referable to stones (scale bar = 100 μm). (c) Detail of a vacuolar degenerations in the tubular epithelial cells (scale bar = 100 μm). (d) Detail at higher magnification of bacterial aggregates in the interstitial haematopoietic tissue of the kidney (asterisks) (scale bar = 50 μm).

The kidney showed diffuse necrosis and tubular ectasia, occasionally with granular content in the lumen, while bacterial aggregates were observed in the interstitium. Additionally, basophilic material consistent with nephrocalcinosis was present (Figure 3b,d). Lymphocytic infiltration of the interstitium and rarefaction of the haematopoietic tissue were also noted. Hyaline droplet and vacuolar degeneration of the tubular epithelial cells were likewise observed (Figure 3c). Histopathological analysis revealed a bacterial septicemia referable to a Photobacteriosis outbreak, showing similar characteristics in all three subjects.

In the present study, we report the isolation of *P. damselae* subsp. *damselae* from diseased common guitarfish, highlighting the wide variety of aquatic animals that this pathogen can infect. As previously observed by Labella et al. (2011), this bacterium has been one of the most frequently occurring pathogens involved in disease outbreaks among farmed fish in Spain. Moreover, in a recent study, Catanese and Grau (2023) described *P. damselae* subsp. *damselae* as a primary pathogen in nursehound shark ( *Scyliorhinus stellaris* ) kept in a confined environment.

This microorganism is ubiquitous in the aquatic environment, which in addition to being potentially zoonotic for humans, can cause disease in marine fish and crustaceans (Matanza and Osorio 2020; Osorio et al. 2018; Rivas et al. 2013). The strains of this pathogen have been isolated in sea and estuarine waters, seaweeds, apparently uninfected marine animals and seafood (Rivas et al. 2013); it is considered a common member of the natural microbiota of healthy carcharhinid sharks (Correia Costa et al. 2022). Furthermore, it is considered a primary pathogen of many wild fish species (damselfish, catfish, shark, stingray, etc.), as well as of fish species of economic importance in aquaculture (turbot, rainbow trout, eel, sea bream, sea bass among others) (Rivas et al. 2013). Infections caused by this bacterium typically result in wound infections and hemorrhagic septicemia, as documented in several cases, including the present study (Catanese and Grau 2023; Rivas et al. 2013).

Regarding the histological findings, lesions in the kidney, like vacuolar degenerations and lymphocyte infiltration, as well as congestion in the liver and gills were previously described in the literature, not only in sharks (Catanese and Grau 2023; Shao et al. 2019).

Identifying the source of transmission and detecting the presence of this pathogen in an aquarium tank pose significant challenges, and several hypotheses can be proposed. In general, many wild fish struggle to adapt to artificial conditions, leading to potential disease outbreaks in aquariums (Smith et al. 2004).

The pathogenic potential of *P. damselae* subsp. *damselae* is well established. It acts both as a free‐living bacterium and as a pathogen with haemolytic and cytolytic activities, affecting a wide range of animal taxa (Osorio et al. 2018).

Nonetheless, it cannot be ruled out that the bacteria might have been present at the time of capture as part of the normal intestinal flora (Correia Costa et al. 2022), and a stress‐inducing event associated with the confined environment care may have triggered the overt infection.

Over the past few decades, various vaccine formulations have been developed for certain aquacultured fish species against Photobacteriosis, and some vaccines are already available on the market (Su and Chen 2022). Consequently, further research into the formulation and implementation of new vaccines for this shark species, as well as the development of effective vaccination strategies in exhibition aquaria, could represent a future challenge to proactively manage this disease in the context of species conservation. Additionally, implementing proper hygiene protocols related to husbandry, equipment, animal health monitoring and handling can help reduce stress on captive individuals and mitigate the risk of infections.

## Author Contributions

**Giorgia Bignami:** methodology, writing – original draft preparation, writing – review and editing. **Teresa Pirollo:** methodology, formal analysis, writing – original draft preparation, writing – review and editing. **Perla Tedesco:** methodology, writing – review and editing. **Monica Caffara:** conceptualization, writing – review and editing. **Maria Letizia Fioravanti:** conceptualization, methodology, writing – review and editing. **Elena Campesi:** sample and anamnestic data provider. **Cristiano Da Rugna:** methodology, sample provider. **Stefano Gridelli:** methodology, sample provider. **Francesco Quaglio:** methodology, writing – review and editing. **Andrea Gustinelli:** conceptualization, writing – original draft preparation, methodology, writing – review and editing, supervision.

## Conflicts of Interest

The authors declare no conflicts of interest.

## Data Availability

The data that support the findings of this study are available from the corresponding author upon reasonable request.
